# *tert*-Butyl 2-(dihydroxyboryl)pyrrole-1-carboxylate

**DOI:** 10.1107/S1600536808013482

**Published:** 2008-05-10

**Authors:** Tomasz Klis, Janusz Serwatowski

**Affiliations:** a Warsaw University of Technology, Faculty of Chemistry, Noakowskiego 3, 00-664, Warsaw, Poland

## Abstract

In the title compound, C_9_H_14_BNO_4_, the carbonyl and boronic acid groups are essentially coplanar with the pyrrole ring and the boronic acid group has an *exo*-*endo* conformation. The *exo*-oriented OH is engaged in an intra­molecular O—H⋯O inter­action, while the *endo*-oriented one is involved in inter­molecular hydrogen bonding to form centrosymmetric dimers. A supra­molecular assembly is achieved through inter­actions involving the *tert*-butyl groups, forming infinite chains along the crystallographic *b* axis. There are, in addition, face-to-face and center-to-edge stacking inter­actions [distance between the pyrrole ring centroid and an N atom from a neighbouring mol­ecule = 3.369 (8) Å].

## Related literature

For related literature, see: Dabrowski *et al.* (2006 ▶); Parry *et al.* (2002 ▶); Saygili *et al.* (2004 ▶); Seminario *et al.* (1998 ▶); Thompson *et al.* (2005 ▶); Wang *et al.* (2002 ▶).
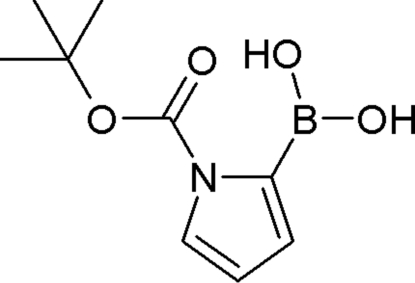


## Experimental

### 

#### Crystal data

C_9_H_14_BNO_4_
*M* *_r_* = 211.02
Orthorhombic, 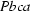

*a* = 12.9179 (12) Å
*b* = 9.5885 (7) Å
*c* = 17.5811 (15) Å
*V* = 2177.7 (3) Å^3^
*Z* = 8
Mo *K*α radiation
μ = 0.10 mm^−1^
*T* = 100 (2) K
0.71 × 0.34 × 0.22 mm

#### Data collection

Kuma KM4 CCD diffractometer
Absorption correction: multi-scan (*CrysAlis RED*; Oxford Diffraction 2005 ▶) *T*_min_ = 0.95, *T*_max_ = 0.98
19290 measured reflections
2713 independent reflections
1911 reflections with *I* > 2σ(*I*)
*R*_int_ = 0.021

#### Refinement

*R*[*F* ^2^ > 2σ(*F* ^2^)] = 0.031
*wR*(*F* ^2^) = 0.076
*S* = 0.96
2713 reflections
193 parameters
All H-atom parameters refined
Δρ_max_ = 0.26 e Å^−3^
Δρ_min_ = −0.19 e Å^−3^

### 

Data collection: *CrysAlis CCD* (Oxford Diffraction, 2005 ▶); cell refinement: *CrysAlis RED* (Oxford Diffraction, 2005 ▶); data reduction: *CrysAlis RED*; program(s) used to solve structure: *SHELXS97* (Sheldrick, 2008 ▶); program(s) used to refine structure: *SHELXL97* (Sheldrick, 2008 ▶); molecular graphics: *DIAMOND* (Brandenburg, 1999 ▶); software used to prepare material for publication: *SHELXL97*.

## Supplementary Material

Crystal structure: contains datablocks I, New_Global_Publ_Block. DOI: 10.1107/S1600536808013482/bg2173sup1.cif

Structure factors: contains datablocks I. DOI: 10.1107/S1600536808013482/bg2173Isup2.hkl

Additional supplementary materials:  crystallographic information; 3D view; checkCIF report

## Figures and Tables

**Table 1:** Hydrogen-bond geometry (Å, °)

*D*—H⋯*A*	*D*—H	H⋯*A*	*D*⋯*A*	*D*—H⋯*A*
O1—H1*O*⋯O3	0.917 (15)	1.704 (15)	2.5941 (10)	162.9 (13)
O2—H2*O*⋯O1^i^	0.922 (16)	1.855 (17)	2.7728 (11)	173.7 (13)

Symmetry code: (i) 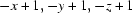.

**Fig. 1.**
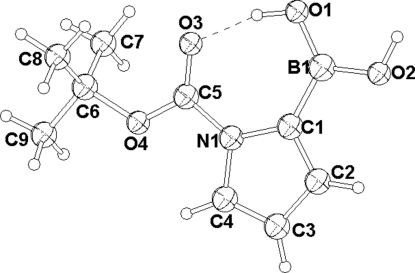
The molecular structure of (I), showing the atom-labelling scheme. Displacement ellipsoids for all non-H atoms are drawn at the 50% probability level.

**Fig. 2.**
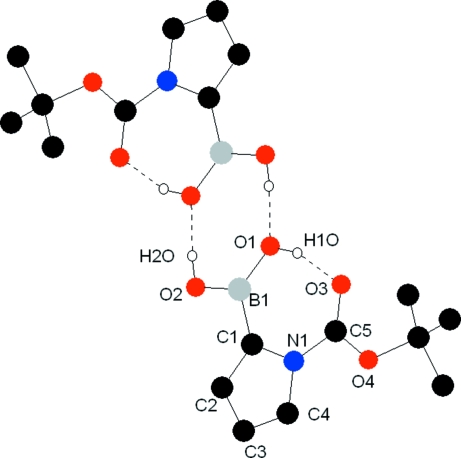
The hydrogen-bonding pattern for (I). Hydrogen bonds are shown as dashed lines.

**Fig. 3.**
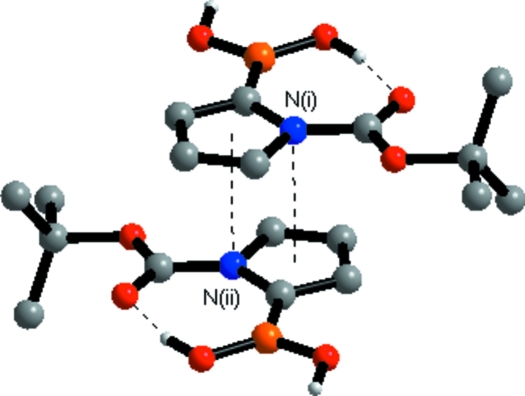
The crystal packing for (I), showing π-π interactions as dotted lines [Symmetry codes: (i) -1/2 + x, 1.5 - y, 1 - z; (ii) 0.5 - x, -1/2 + y, z].

## supplementary crystallographic information

### Comment

Nitrogen-containing boronic acids are the object of interest to many chemists because of their application as potential saccharide sensors (Wang *et al.*, 2002). However, no crystal structure of any boronic acid containing a pyrrole ring has been elucidated to date. The only crystal data concerning nitrogen-containing heterocyclic boronic acids involve some pyrydine (Parry *et al.*, 2002), (Thompson *et al.*, 2005), (Dabrowski *et al.*, 2006) and pyrimidine (Saygili *et al.*, 2004) derivatives.

The molecular structure of the title compound  C_9_H_14_BO_4_N (I) is shown in Fig. 1. The carbonyl and boronic acid groups are essentially coplanar with the pyrrole ring [torsion angles O3—C5—N1—C1 = -1.31 (1)° and N1—C1—B1—O1 = -2.8 (2)° respectively]. The conformation between C9 from the *tert*-butyl- and the carbonyl groups is antiperiplanar. The boronic acid group has an *exo*-*endo* conformation. The *exo*-oriented OH is engaged in an intramolecular O—H···O interaction with O3. The *endo*- oriented one, instead, is involved into intermolecular hydrogen bonding to form centrosymmetric dimers (Fig. 2). The supramolecular assembly is achieved through interactions involving *tert*-butyl groups, forming infinite chains along the crystallographic *b* axis. Examination of the crystal packing reveals the presence of face to face, center to edge stacking (FFCE) (Seminario *et al.*, 1998). These interactions are represented by a relatively short distance (3.369 (8) Å) between the pyrrole ring centroid and the nitrogen atom from neighbouring molecules (Fig. 3).

### Experimental

N-tert-butoxycarbonyl-pyrrole-2-boronic acid was obtained from Aldrich, crystallized from tetrahydrofurane and dried in air.

### Refinement

All of hydrogen atoms were located geometrically and their positions were refined while temperature factors were not. The maximum electron-density peak in the final difference map is 0.80 Å from atom C1.

### Crystal data

**Table:**

C_9_H_14_BNO_4_	*F*_000_ = 896
*M**_r_* = 211.02	*D*_x_ = 1.287 Mg m^−^^3^
Orthorhombic, *P**b**c**a*	Mo *K*α radiation λ = 0.71073 Å
Hall symbol: -P 2ac 2ab	Cell parameters from 19290 reflections
*a* = 12.9179 (12) Å	θ = 2.8–28.7º
*b* = 9.5885 (7) Å	µ = 0.10 mm^−^^1^
*c* = 17.5811 (15) Å	*T* = 100 (2) K
*V* = 2177.7 (3) Å^3^	Prismatic, colourless
*Z* = 8	0.71 × 0.34 × 0.22 mm

### Data collection

**Table:**

Kuma KM4 CCD diffractometer	2713 independent reflections
Radiation source: fine-focus sealed tube	1911 reflections with *I* > 2σ(*I*)
Monochromator: graphite	*R*_int_ = 0.021
Detector resolution: 8.6479 pixels mm^-1^	θ_max_ = 28.7º
*T* = 100(2) K	θ_min_ = 2.8º
ω scan	*h* = −17→17
Absorption correction: multi-scan(CrysAlis RED; Oxford Diffraction 2005)	*k* = −12→12
*T*_min_ = 0.95, *T*_max_ = 0.98	*l* = −23→23
19290 measured reflections	

### Refinement

**Table:**

Refinement on *F*^2^	Hydrogen site location: inferred from neighbouring sites
Least-squares matrix: full	All H-atom parameters refined
*R*[*F*^2^ > 2σ(*F*^2^)] = 0.031	*w* = 1/[σ^2^(*F*_o_^2^) + (0.0461*P*)^2^] where *P* = (*F*_o_^2^ + 2*F*_c_^2^)/3
*wR*(*F*^2^) = 0.076	(Δ/σ)_max_ = 0.001
*S* = 0.97	Δρ_max_ = 0.26 e Å^−^^3^
2713 reflections	Δρ_min_ = −0.19 e Å^−^^3^
193 parameters	Extinction correction: SHELXL, Fc^*^=kFc[1+0.001xFc^2^λ^3^/sin(2θ)]^-1/4^
Primary atom site location: structure-invariant direct methods	Extinction coefficient: 0.0033 (7)
Secondary atom site location: difference Fourier map	

### Special details

**Table:**

Geometry. All e.s.d.'s (except the e.s.d. in the dihedral angle between two l.s. planes) are estimated using the full covariance matrix. The cell e.s.d.'s are taken into account individually in the estimation of e.s.d.'s in distances, angles and torsion angles; correlations between e.s.d.'s in cell parameters are only used when they are defined by crystal symmetry. An approximate (isotropic) treatment of cell e.s.d.'s is used for estimating e.s.d.'s involving l.s. planes.
Refinement. Refinement of *F*^2^ against ALL reflections. The weighted *R*-factor *wR* and goodness of fit *S* are based on *F*^2^, conventional *R*-factors *R* are based on *F*, with *F* set to zero for negative *F*^2^. The threshold expression of *F*^2^ > σ(*F*^2^) is used only for calculating *R*-factors(gt) *etc*. and is not relevant to the choice of reflections for refinement. *R*-factors based on *F*^2^ are statistically about twice as large as those based on *F*, and *R*- factors based on ALL data will be even larger.

### Fractional atomic coordinates and isotropic or equivalent isotropic displacement parameters (Å^2^)

**Table:**

	*x*	*y*	*z*	*U*_iso_*/*U*_eq_	
O1	0.54350 (6)	0.63771 (7)	0.45212 (4)	0.02586 (19)	
O2	0.40336 (6)	0.64180 (8)	0.53731 (4)	0.02619 (19)	
O3	0.60617 (5)	0.83554 (7)	0.36263 (4)	0.02260 (18)	
O4	0.56150 (5)	1.04637 (7)	0.31656 (4)	0.02186 (18)	
N1	0.46192 (6)	0.95162 (8)	0.40642 (4)	0.01800 (19)	
C1	0.42176 (8)	0.85423 (10)	0.45945 (5)	0.0187 (2)	
C2	0.33504 (8)	0.91604 (11)	0.48868 (6)	0.0223 (2)	
C3	0.32126 (8)	1.05007 (11)	0.45569 (6)	0.0242 (2)	
C4	0.39900 (8)	1.06957 (10)	0.40571 (6)	0.0218 (2)	
C5	0.54985 (8)	0.93691 (9)	0.36119 (5)	0.0186 (2)	
C6	0.64575 (8)	1.04984 (10)	0.25803 (5)	0.0225 (2)	
C7	0.63032 (10)	0.93008 (12)	0.20274 (6)	0.0285 (3)	
C8	0.75028 (9)	1.04896 (12)	0.29709 (6)	0.0269 (2)	
C9	0.62477 (10)	1.18889 (12)	0.22011 (7)	0.0304 (3)	
B1	0.46066 (9)	0.70564 (12)	0.48297 (6)	0.0204 (2)	
H1O	0.5763 (11)	0.6964 (15)	0.4187 (8)	0.056 (4)*	
H2O	0.4234 (12)	0.5504 (17)	0.5442 (8)	0.060 (5)*	
H2	0.2915 (8)	0.8741 (11)	0.5269 (5)	0.018 (2)*	
H3	0.2680 (9)	1.1122 (12)	0.4668 (6)	0.028 (3)*	
H4	0.4168 (8)	1.1463 (11)	0.3729 (6)	0.020 (3)*	
H7A	0.5580 (11)	0.9321 (12)	0.1830 (7)	0.038 (3)*	
H7B	0.6429 (9)	0.8387 (12)	0.2253 (6)	0.029 (3)*	
H7C	0.6788 (9)	0.9424 (11)	0.1603 (6)	0.029 (3)*	
H8A	0.7576 (9)	1.1342 (13)	0.3317 (7)	0.038 (3)*	
H8B	0.7629 (9)	0.9613 (12)	0.3252 (6)	0.031 (3)*	
H8C	0.8046 (10)	1.0547 (12)	0.2579 (7)	0.036 (3)*	
H9A	0.6288 (8)	1.2649 (12)	0.2567 (7)	0.034 (3)*	
H9B	0.5551 (10)	1.1878 (13)	0.1981 (6)	0.040 (3)*	
H9C	0.6768 (9)	1.2037 (12)	0.1802 (7)	0.039 (3)*	

### Atomic displacement parameters (Å^2^)

**Table:**

	*U*^11^	*U*^22^	*U*^33^	*U*^12^	*U*^13^	*U*^23^
O1	0.0238 (4)	0.0218 (4)	0.0320 (4)	0.0014 (3)	0.0056 (3)	0.0085 (3)
O2	0.0255 (4)	0.0253 (4)	0.0278 (4)	−0.0001 (3)	0.0051 (3)	0.0059 (3)
O3	0.0227 (4)	0.0186 (4)	0.0265 (4)	0.0021 (3)	0.0044 (3)	0.0032 (3)
O4	0.0240 (4)	0.0180 (4)	0.0235 (4)	0.0007 (3)	0.0031 (3)	0.0038 (3)
N1	0.0172 (4)	0.0178 (4)	0.0190 (4)	0.0001 (3)	−0.0011 (3)	−0.0005 (3)
C1	0.0181 (5)	0.0220 (5)	0.0159 (4)	−0.0040 (4)	−0.0023 (4)	−0.0013 (4)
C2	0.0188 (5)	0.0286 (6)	0.0194 (5)	−0.0018 (4)	−0.0009 (4)	−0.0039 (4)
C3	0.0195 (5)	0.0264 (6)	0.0267 (5)	0.0049 (5)	−0.0040 (4)	−0.0071 (4)
C4	0.0220 (5)	0.0194 (5)	0.0239 (5)	0.0029 (4)	−0.0061 (4)	−0.0022 (4)
C5	0.0203 (5)	0.0170 (5)	0.0186 (5)	−0.0027 (4)	−0.0026 (4)	−0.0011 (4)
C6	0.0245 (6)	0.0228 (5)	0.0203 (5)	−0.0023 (4)	0.0036 (4)	0.0031 (4)
C7	0.0341 (7)	0.0281 (6)	0.0233 (6)	−0.0037 (5)	0.0027 (5)	−0.0002 (4)
C8	0.0258 (6)	0.0270 (6)	0.0278 (6)	−0.0045 (5)	0.0011 (5)	0.0047 (5)
C9	0.0339 (7)	0.0270 (6)	0.0304 (6)	−0.0027 (5)	0.0012 (5)	0.0087 (5)
B1	0.0193 (6)	0.0230 (6)	0.0190 (5)	−0.0038 (5)	−0.0027 (5)	−0.0013 (5)

### Geometric parameters (Å, °)

**Table:**

O1—B1	1.3652 (13)	C3—H3	0.931 (12)
O1—H1O	0.917 (15)	C4—H4	0.963 (10)
O2—B1	1.3547 (13)	C6—C8	1.5149 (15)
O2—H2O	0.922 (16)	C6—C9	1.5151 (14)
O3—C5	1.2143 (11)	C6—C7	1.5176 (14)
O4—C5	1.3191 (11)	C7—H7A	0.996 (13)
O4—C6	1.4981 (12)	C7—H7B	0.976 (11)
N1—C4	1.3928 (12)	C7—H7C	0.981 (12)
N1—C5	1.3937 (12)	C8—H8A	1.023 (13)
N1—C1	1.4178 (12)	C8—H8B	0.989 (12)
C1—C2	1.3676 (14)	C8—H8C	0.985 (13)
C1—B1	1.5665 (15)	C9—H9A	0.973 (12)
C2—C3	1.4211 (15)	C9—H9B	0.980 (13)
C2—H2	0.964 (10)	C9—H9C	0.983 (12)
C3—C4	1.3474 (15)		
			
B1—O1—H1O	108.9 (9)	O4—C6—C7	109.13 (8)
B1—O2—H2O	111.6 (9)	C8—C6—C7	113.78 (9)
C5—O4—C6	120.61 (7)	C9—C6—C7	111.13 (9)
C4—N1—C5	123.55 (8)	C6—C7—H7A	109.4 (7)
C4—N1—C1	109.10 (8)	C6—C7—H7B	113.4 (6)
C5—N1—C1	127.35 (8)	H7A—C7—H7B	108.4 (10)
C2—C1—N1	105.15 (8)	C6—C7—H7C	108.2 (6)
C2—C1—B1	123.90 (9)	H7A—C7—H7C	109.3 (9)
N1—C1—B1	130.94 (9)	H7B—C7—H7C	108.1 (9)
C1—C2—C3	109.95 (9)	C6—C8—H8A	110.3 (7)
C1—C2—H2	124.0 (6)	C6—C8—H8B	112.2 (7)
C3—C2—H2	126.0 (6)	H8A—C8—H8B	111.5 (9)
C4—C3—C2	107.35 (9)	C6—C8—H8C	108.5 (7)
C4—C3—H3	126.8 (7)	H8A—C8—H8C	107.8 (9)
C2—C3—H3	125.9 (7)	H8B—C8—H8C	106.2 (9)
C3—C4—N1	108.44 (9)	C6—C9—H9A	111.0 (7)
C3—C4—H4	132.4 (6)	C6—C9—H9B	109.2 (7)
N1—C4—H4	119.1 (6)	H9A—C9—H9B	108.6 (10)
O3—C5—O4	125.51 (9)	C6—C9—H9C	108.6 (7)
O3—C5—N1	123.88 (8)	H9A—C9—H9C	109.1 (10)
O4—C5—N1	110.60 (8)	H9B—C9—H9C	110.4 (9)
O4—C6—C8	109.64 (8)	O2—B1—O1	119.54 (10)
O4—C6—C9	101.06 (8)	O2—B1—C1	114.96 (9)
C8—C6—C9	111.34 (9)	O1—B1—C1	125.49 (9)
			
C4—N1—C1—C2	0.54 (10)	C4—N1—C5—O3	178.49 (9)
C5—N1—C1—C2	−179.63 (8)	C1—N1—C5—O3	−1.32 (14)
C4—N1—C1—B1	179.79 (9)	C4—N1—C5—O4	−2.55 (12)
C5—N1—C1—B1	−0.38 (15)	C1—N1—C5—O4	177.64 (8)
N1—C1—C2—C3	−0.70 (10)	C5—O4—C6—C8	−64.93 (11)
B1—C1—C2—C3	179.99 (9)	C5—O4—C6—C9	177.47 (8)
C1—C2—C3—C4	0.62 (11)	C5—O4—C6—C7	60.31 (11)
C2—C3—C4—N1	−0.26 (11)	C2—C1—B1—O2	−1.97 (14)
C5—N1—C4—C3	179.99 (8)	N1—C1—B1—O2	178.91 (9)
C1—N1—C4—C3	−0.17 (10)	C2—C1—B1—O1	176.28 (10)
C6—O4—C5—O3	3.95 (14)	N1—C1—B1—O1	−2.85 (16)
C6—O4—C5—N1	−174.99 (7)		

### Hydrogen-bond geometry (Å, °)

**Table:**

*D*—H···*A*	*D*—H	H···*A*	*D*···*A*	*D*—H···*A*
O1—H1O···O3	0.917 (15)	1.704 (15)	2.5941 (10)	162.9 (13)
O2—H2O···O1^i^	0.922 (16)	1.855 (17)	2.7728 (11)	173.7 (13)

Symmetry codes: (i) −*x*+1, −*y*+1, −*z*+1.
